# Galanin receptor ligands

**DOI:** 10.1186/2193-1801-4-S1-L18

**Published:** 2015-06-12

**Authors:** Ülo Langel

**Affiliations:** Stockholm University, Sweden

**Keywords:** Galanin, neuropeptide, ligand

The effect of galanin is mediated through three GPCR subtypes, GalR1-3. The limited number of specific ligands to the galanin receptor subtypes has hindered the understanding of the individual effects of each receptor subtype. This review summarizes the current data of the importance of the galanin receptor subtypes and receptor subtype specific agonists and antagonists and their involvement in different biological and pathological functions.

